# Intake of Fat-Soluble Vitamins in the Belgian Population: Adequacy and Contribution of Foods, Fortified Foods and Supplements

**DOI:** 10.3390/nu9080860

**Published:** 2017-08-11

**Authors:** Isabelle Moyersoen, Brecht Devleesschauwer, Arnold Dekkers, Karin de Ridder, Jean Tafforeau, John van Camp, Herman van Oyen, Carl Lachat

**Affiliations:** 1 Department of Public Health and Surveillance, Scientific Institute of Public Health (WIV-ISP), Juliette Wytsmanstraat 14, 1050 Brussels, Belgium; brecht.devleesschauwer@wiv-isp.be (B.D.); Karin.DeRidder@wiv-isp.be (K.d.R.); Jean.Tafforeau@wiv-isp.be (J.T.); herman.vanoyen@wiv-isp.be (H.v.O.); 2 Department of Food Safety and Food Quality, Ghent University, Coupure links 653, 9000 Ghent, Belgium; john.vancamp@ugent.be (J.v.C.); Carl.lachat@Ugent.be (C.L.); 3 Department for Statistics, Informatics and Modelling, National Institute for Public Health and the Environment (RIVM), P.O. BOX 1, 3720 BA Bilthoven, The Netherlands; arnold.dekkers@rivm.nl; 4 Department of Public Health, Ghent University, De Pintelaan 185, 9000 Gent, Belgium

**Keywords:** dietary intake, micronutrient adequacy, fat-soluble vitamins, fortified foods, supplements, Belgian population

## Abstract

A key challenge of public health nutrition is to provide the majority of the population with a sufficient level of micronutrients while preventing high-consumers from exceeding the tolerable upper intake level. Data of the 2014 Belgian food consumption survey (*n* = 3200) were used to assess fat-soluble vitamin (vitamins A, D, E and K) intake from the consumption of foods, fortified foods and supplements. This study revealed inadequate intakes for vitamin A, from all sources, in the entire Belgian population and possible inadequacies for vitamin D. The prevalence of inadequate intake of vitamin A was lowest in children aged 3–6 (6–7%) and highest in adolescents (girls, 26%; boys, 34–37%). Except for women aged 60–64 years, more than 95% of the subjects had vitamin D intake from all sources below the adequate intake (AI) of 15 μg/day. The risk for inadequate intake of vitamins K and E was low (median > AI). Belgian fortification and supplementation practices are currently inadequate to eradicate suboptimal intakes of vitamins A and D, but increase median vitamin E intake close to the adequate intake. For vitamin A, a small proportion (1–4%) of young children were at risk of exceeding the upper intake level (UL), while for vitamin D, inclusion of supplements slightly increased the risk for excessive intakes (% > UL) in adult women and young children. The results may guide health authorities when developing population health interventions and regulations to ensure adequate intake of fat-soluble vitamins in Belgium.

## 1. Introduction

Fat-soluble vitamins are indispensable to ascertain optimum health in all life stages [1]. Except for vitamin D, which is mainly produced under the influence of ultraviolet B (UVB) radiation on the skin, dietary intake is the main source for all vitamins. A well-balanced and healthy diet is therefore essential to prevent disease and reduce the burden of disease [2].

Over the last few decades, diets in high income countries have shifted to more energy-dense and nutrient-poor diets, resulting in increasing prevalence of inadequate intake and suboptimal status of micronutrients [3,4,5]. Inadequate intakes of vitamins A and D are a general concern in different populations and population subgroups in Europe [4,5]. The reduction of the prevalence of micronutrient inadequacies should therefore be a priority in food and nutrition policies in Europe [6]. Adequate intake of micronutrients can be achieved through health promotion, as well as through fortification and supplementation practices [1,7]. In the USA, fortified foods and/or supplements contribute largely to the intake of fat-soluble vitamins and help achieve the recommended intakes for vitamins A, D and E for some part, but not all of the population [8,9]. However, while fortified foods are well adopted in the USA, Europeans are more skeptical of fortification [10]. In Europe, non-fortified foods are the main source for most vitamins. Despite a uniform regularization within the EU, fortification and supplementation practices differ substantially between countries [1].

It has been repeatedly reported in European studies that voluntarily-fortified foods can have a significant impact on reducing the proportion of inadequate intakes within a population [1,11,12]. Supplements generally have a more important contribution to total vitamin intake than voluntarily-fortified foods, but are often less effective at reducing the proportion of suboptimal intakes due to insufficient adherence [3,12]. In response to very low intakes (e.g., vitamin D), national encouraged fortification can play an important role in the reduction of inadequacies. In Finland, vitamin D fortification of dairy products and fats was successfully introduced as a strategy to improve vitamin D status in the entire population [13].

Despite the positive effects of fortified foods and supplements, excessive intake of fat-soluble vitamins can lead to adverse health effects [14]. In general, however, only a small proportion of the population, mainly children, exceeds the upper intake level (UL) [1,8]. A general concern of excessive intakes remains when for certain nutrients, both fortification and supplementation practices are common [1,3,15]. Vitamins with a relative low UL in comparison to the recommended intakes, like vitamins A, D and E, need special attention [3].

In Belgium, fortified foods and supplements require notification, and the level of vitamins added is governed by the Belgian Royal Decree (Koninklijk besluit (KB) 3/3/1992). Fortification in Belgium is mandatory for margarines (fat content (FC) > 80%) and spreadable fats (39% < FC < 41%) (KB 02/10/1980) and officially encouraged for spreadable fats with other fat contents. Nutritive fortification values of margarines and spreadable fats however are currently based on nutritive values of butter (Table 1).

Similar to other countries, the Belgian Superior Health Council advises a vitamin D supplement for the entire population in the case of minimal exposure to sunlight [15,16]. Independently of their intake from food, a supplementation of 10 μg/day for children and 15 μg/day for adolescents and adults is recommended. This supplementation advice however is currently based on the population reference intake. Dietary intake data are needed to fine-tune these recommendations in correspondence with Belgian dietary habits, as well as with intake from fortified foods and supplements [17].

When designing strategies to combat inadequate or excessive intakes, it is important to evaluate the efficacy of the actual fortification and supplementation programs. Knowledge of the intake distribution of fat-soluble vitamins and the contribution of all sources is therefore required [1,3,8]. In Belgium, those studies are scarce. One study evaluated the intake of micronutrients in Flemish preschool children (2.5–6.5 years). Unfortunately analyses of fat-soluble vitamins were confined to vitamin D, and dietary supplements were hereby not taken into account [18]. Nevertheless, in the review study of Mensink et al., raw data from eight countries were uniformly re-analyzed to evaluate inadequacies of micronutrients in Europe. This study included results for vitamin A, D and E from the Flemish children study [4].

The current study aims to evaluate the contribution of mandatorily fortified foods, voluntarily fortified foods and supplements to the intake of fat-soluble vitamins in the general Belgian population (3–64 years) and to compare usual intake to dietary reference values. As food consumption varies by age and gender, the intake distributions were stratified by age and gender.

## 2. Materials and Methods 

### 2.1. Data Collection

Data of the Belgian food consumption survey (BFCS) 2014 were used to assess the intake of fat-soluble vitamins in the Belgian population. The survey is described in detail by Bel et al. [19]. In brief, data were collected among a representative sample of the Belgian population (*n* = 3200, 3–64 years old) following a multistage stratified sampling design. This included a geographical stratification according to the provinces (10 Belgian provinces and the Brussels Capital Region), a selection of municipalities within each geographical stratum and a selection of individuals within each municipality. The national population register was used as the sampling frame. The sample was stratified into five sex-age groups, i.e., 3–5, 6–9, 10–17, 18–39 and 40–64 years [20]. Seasonal effects and day-to-day variation in food consumption were taken into account by equally dividing the interviews between all weekdays and seasons.

Food and supplement consumption data in adults and adolescents (10–64 years old) were collected by a dietician using two non-consecutive 24-h dietary recalls (using the GloboDiet^®^ software) and a self-administered food frequency questionnaire (FFQ) [20]. In children (3–9 years old), consumption data were collected using two non-consecutive one-day food diaries, a completion interview by a dietician using GloboDiet^®^ and a self-administered frequency questionnaire. Data in children were collected through their proxy (i.e., one of the parents or legal guardians). The self-administered frequency questionnaire included questions on supplements’ consumption with a recall period of one year.

In all subjects, socio-demographic characteristics were collected by interview, while height, weight and waist circumference were measured by the dietician during the second home-visit. The participation rate of the survey was 37%. Weighting factors were used to ensure the representativeness of the sample for age, gender and place of residency [19].

All participants or their proxies provided written informed consent. Approvals of the Ethics Committee of Ghent University and the Commission for the Protection of Privacy were obtained. The study was conducted in accordance with the ethical principles for medical research involving human subjects [19].

### 2.2. Food Composition

#### 2.2.1. Original Food Composition Table

Fat-soluble vitamin intake was calculated by combining the consumption data of the BFCS 2014 with food and supplement composition data. The latter were retrieved from the Belgian food composition table NUBEL and the Dutch composition table NEVO [19,21,22]. Data on supplement composition were obtained either from Farmacompendium 2014 [23] (an online database for pharmaceutical products in Belgium), or from label information, or the Internet.

#### 2.2.2. Adapted Food Composition Table

Fortified foods are subject to permanent modifications in terms of composition and brands [24]. Furthermore, food codes in national food composition tables are often not brand-specific and therefore lacking information on fortified nutrient content. At the beginning of 2015, a market research was conducted to make an inventory of the actual supply of fortified foods on the Belgian market. This inventory was done in supermarkets and grocery stores by physical visits, by means of supermarket websites and via manufacturers’ websites. The fortified nutrient content as labelled on the packages was obtained for all foods fortified with vitamins A, D, E and/or K. This information, however, does not differentiate between the naturally-occurring amount and the amount added during fortification. The nutrient content on the label hence reflects the total content of the fortified nutrient [3].

In case of missing data, four extra national food composition tables were used in the following order: (1) McCance and Widowson’s, the U.K. food composition table [25]; (2) Ciqual, the French composition table [26]; (3) the Danish food composition table [27] and (4) the American food composition table [28]. When the missing vitamin value was not found in the first source, the next source was consulted till the appropriate information was retrieved. When data were still missing, values were retrieved from ingredient-based or recipe-based calculations, through similarities or as a median value [29]. This was especially the case for vitamin K and D. More details on the market research and the compilation of the adapted food composition table can be found elsewhere [30].

## 3. Statistical Analysis

### 3.1. Contribution of Food Groups

Contributions of food groups to total vitamins A, D, E or K intake were calculated following the GloboDiet^®^ classification system based on 18 food groups (Supplementary Table S1). Calculations were based on the first 24-h recall. For each subject, the proportion of vitamin intake between the respective food group and the total vitamin intake was calculated. The mean contribution of a specific food group to the total vitamin intake of the Belgian population was calculated as the survey-weighted mean of these individual proportions [31]. Analyses were conducted in SAS 9.3 (SAS Institute Inc., Cary, NC, USA).

### 3.2. Intake Modelling

The Statistical Program to Assess Dietary Exposure (SPADE) was used to estimate the habitual intake distributions for vitamins A, D, E and K from all sources. SPADE.RIVM is a freely available R package with different modelling options [32]. The multipart model was used, which allowed estimating the habitual intake from food, mandatorily fortified foods, voluntarily fortified food and supplements separately. At the end, the habitual amounts were added to the habitual intake. Specific challenges such as multimodality, a spiked distribution of vitamin dosages in supplements and heterogeneity in variances due to the differing intakes (daily versus episodically) from foods versus fortified foods and supplements were taken into account [32]. SPADE further accounts for the within-person variability typical for short-term assessment methods like the 24-h recall, by means of a “first shrink then add” method.

Intake data were modelled as a function of age and reported in the age ranges used by the European Food Safety Authority (EFSA) in setting their dietary reference values, i.e., children 3–6 and 7–10, adolescents 11–14 and 15–17 and adults 18–39 and 40–64 years. The 5th, 50th, 95th and 97.5th percentiles, as well as the arithmetic mean intakes of vitamins A, D, E and K were calculated for foods only, foods plus fortified foods and foods plus fortified foods plus supplements. For vitamins A and D for which mandatory fortification is applied on margarines and spreadable fats, an additional intake from mandatorily fortified foods was calculated. Uncertainty of mean intake is quantified with bootstrap 95% confidence intervals [32].

For the purpose of this study, supplement users were defined as subjects that indicated the use of supplements on one of the 24-h recalls or on the FFQ. Subjects that had not completed the FFQ (*n* = 900) were treated as users when they consumed a supplement on one of the 24-h recalls or never-users when they did not consume supplements on both 24-h recalls. Supplements comprised single vitamins, multivitamins or mineral-vitamin supplements containing the respective vitamin.

### 3.3. Adequacy of Vitamin Intake

To evaluate the adequacy of the intake of fat-soluble vitamins, the intake distribution of the Belgian population was evaluated against the dietary reference values set by EFSA [14,33,34,35,36].

For vitamin A and retinol, insufficient intakes were evaluated with the estimated average requirement (EAR) as cut-off value. The EAR is defined as an intake level adequate for 50% of the population [37]. The risk of insufficient intakes in the population was determined as the proportion of the population with usual vitamin A intake below the EAR. Vitamin A intake was expressed in microgram retinol equivalents (μg RE), following the conversion factors adopted by EFSA, i.e., 1 μg RE equals 1 μg of retinol, 6 μg of β-carotene and 12 μg of other pro-vitamin A carotenoids [35]. In this study, only retinol and β-carotene were considered [35].

The reported vitamin K intake comprises total vitamin K intake (vitamin K1 + K2) since some composition tables distinguish phylloquinones (vitamin K1) from menaquinones (vitamin K2), while others only report the total vitamin K value. The dietary reference values for vitamin K (adequate intake (AI) and UL) are based on phylloquinones only. Despite phylloquinones being the major form of “total vitamin K”, this implies some uncertainties in the usual vitamin K intake estimation [36].

When the distribution of requirement of a certain vitamin is not known, an AI is used instead of an EAR. The AI value is based on the median daily intake of an apparently healthy population [37]. The prevalence of inadequate intakes evaluated with an AI was stated as “low” if the median intake of the population for the respective vitamin was above the AI. If the median intake was below the AI, the adequacy for a certain vitamin could not be evaluated (no statement) [38].

Possible excessive intakes for all vitamins were estimated as the proportion of the population with usual intakes above the UL [37]. The UL for vitamin A was based on retinol only. Therefore excessive intakes for vitamin A were expressed as the percentage of the population with habitual retinol intake exceeding the UL [14].

### 3.4. Misreporting

Misreporting of dietary intake affects the estimates of both the low and high percentiles of the intake distributions. Misreporting was identified using Goldberg cut-off values with age-specific physical activity level (PAL) [39,40]. Additional analyses were performed with exclusion of under- and over-reporters. These results are presented in the Supplementary files.

## 4. Results

### 4.1. Inventory of Fortified Foods

Table 1 gives an overview of the inventory of foods fortified with vitamins A, D, E and K available on the Belgian market in 2015 and suitable for the target population. The inventory revealed a considerable amount of fortified foods specially designed for children, i.e., growing-up milk, fortified milk, cereals, cacao, fruit juices, spreadable cheese and dairy desserts. Fortification with vitamin E and especially vitamin D was most common.

### 4.2. Supplement Consumption

The consumption of supplements with vitamins A, D, E or K was low in the Belgian population, respectively 16%, 25%, 17% and 4%. Vitamin A, E and K supplements were mostly consumed as multivitamins, while 44% of vitamin D supplements were consumed as a single vitamin [41].

### 4.3. Contribution of Food Groups and Supplements

Table 2 gives an overview of the food groups that contributed most to the total vitamin, A, D, E and K intake.

Food sources of animal origin, as well as fat and oils were important contributors for all fat-soluble vitamin intakes, while vegetables were the major contributors for vitamins A, E and K. The high contribution of fat and oils for vitamins A and D originates from margarines and spreadable fats that are mandatorily fortified in Belgium to levels similar as in butter, i.e., 700–900 μg/100 g for vitamin A and 6–7.5 μg/100 g for vitamin D.

In Belgium, the intake of vitamins A, D, E and K from supplements was generally low and varied from 0.9% (vitamin K) to 5.8% (vitamin D) of total intake.

### 4.4. Vitamin Intake and Adequacy

Table 3, Table 4, Table 5, Table 6, Table 7, Table 8, Table 9 and Table 10 summarize the intake distributions and estimates of inadequacy (% <EAR or AI and excessive intakes % >UL) for vitamins A, D, E and K in each sex-age group of the Belgian population (3–64 years).

#### 4.4.1. Vitamin A

Usual vitamin A intake in the Belgian population from foods varied from 606 μg/day (girls 3–6 years) to 937 μg/day (men 40–64 years). Foods were the greatest contributors to total vitamin A intake. Contributions from “foods only” varied from 83% in adult women (18–39 years) to 91% in boys (7–10 years) (Table 3 and Table 4).

Inadequate intakes for vitamin A (% <EAR) were found in all sex-age groups. When considering foods only, the proportion of the population with inadequate intake ranged from 5–47%.

Mandatorily fortified foods, namely margarines and spreadable fats, were the second largest contributor to usual vitamin A intake and reduced inadequacy proportions by 2–10%.

Only limited amounts of foods were voluntarily fortified with vitamin A. The contribution of voluntarily fortified foods was consequently low and varied from 0.3–5% and had a minor effect on the reduction of the prevalence of inadequate intakes (1–2% reduction).

The contribution of supplements to total vitamin A intake varied from 0.9% in boys (7–10 years) to 10% in adult women (18–39 years), reducing the proportion of the population with inadequate intake up to 4%.

When considering all sources, a substantial proportion of inadequate vitamin A intakes remained in all sex-age groups of the Belgian population. The prevalence of inadequate vitamin A intake was lowest in children (3–6 years), with levels ranging from 1–2% and highest in adolescents, with levels ranging from 26% in adolescent girls and 34–37% in adolescent boys.

The risk of excessive vitamin A intake (based on retinol only [14]) was nil in almost all sex-age groups. In young boys (3–6 years), however, 4% had a risk of excessive intakes from all sources combined. High intakes originated from the consumption of cream pâté (7797 μg/100 g) [22]. The intake distribution from retinol is illustrated in Supplementary Tables S2 and S3.

Excluding miss-reporters did not change the proportion with excessive intakes, but substantially decreased the proportion of subjects with intakes below the EAR. However, the conclusions within age-groups remained the same (Supplementary Tables S4 and S5).

#### 4.4.2. Vitamin D

Usual vitamin D intake from the consumption of “foods only” was generally low in the Belgian population (Table 5 and Table 6). The mean usual vitamin D intake varied from 2.34 μg/day in boys (3–6 years) to 3.71 μg/day in adult men (40–64 years). With the inclusion of vitamin D from fortified foods and especially from supplements, intake levels increased significantly. The mean usual vitamin D intake from all sources was lowest in adolescents aged 11–14 years (4.85 μg/day in boys; 5.6 μg/day in girls) and highest in children aged 3–6 years (11.61 μg/day in boys, 9.3 μg/day in girls) and adult women aged 18–39 and 40–64 years (9.1 μg/day, respectively 13.1 μg/day).

In Belgium, margarines and spreadable fats are mandatorily fortified with vitamin D with levels of vitamin D ranging from 6–7.5 μg/day (Table 1). Mandatorily-fortified foods contributed between 3.2% and 13.9% to total vitamin D intake.

Contribution of voluntarily fortified foods to total vitamin D intake was more important in children and adolescents than in adults (Table 5 and Table 6). In children and adolescents, fortified foods contributed in a range from 6.6–14.8%. In adults, the contribution of voluntarily fortified foods ranged from 2.7–5.7%.

The contribution of supplements to the total vitamin D intake was considerable. Contribution levels were generally higher in women than in men and ranged from 16.7% in adolescent boys (11–14 years) to 71% in women (40–64 years). At the 97.5th percentiles, the increases in vitamin D levels were most pronounced, indicating high concentrations of vitamin D in supplements.

In all sex-age groups, the median vitamin D intake from “foods only” was below the AI. Despite their important contribution to the mean usual intake, inclusion of fortified foods and supplements only slightly increased median vitamin D intake to levels around 4 μg/day and remained thus far below the AI (13–31% of the AI). Therefore, no statement was made on the adequacy of vitamin D.

The risk of excessive intakes of vitamin D from the consumption of food was nil also when mandatorily and voluntarily fortified foods were considered. With the addition of supplements, the risk of excessive intake remained null, except in children and adult women. 2.5% of boys and 1.7% of girls aged 3–6 years, 1.1% of girls aged 7–10 and 1.4–2% of adult women ages 18–39, respectively 40–64 years had a risk of exceeding the UL for vitamin D.

The median intake of vitamin D hardly changed and remained below the AI when miss-reporters were excluded. The proportion of the population with excessive intakes did not change substantially (Supplementary Tables S6 and S7).

#### 4.4.3. Vitamin E

Usual vitamin E intake from “foods only” in the Belgian population ranged from 7 mg/day in girls (3–6 years) to 13 mg/day in men (18–39 years) (Table 7 and Table 8). The addition of fortified foods and supplements increased the mean intake of vitamin E from levels of 8 mg/day in girls (3–6 years) to 14 mg/100 g in men (18–39 years).

Food was the major contributor to total vitamin E intake. Contribution of “foods only” ranged from 80–92%. Fortified foods had a low contribution to total vitamin E intake. Contributions varied from 4% in adult men (18–39 years) to 15% in boys (3–6 years). The contribution of supplements to total vitamin E intake was generally low with contributions between 2% and 6% except for adult women, where the contribution of supplements was more important and varied from 10–14%.

The median vitamin E intake of foods only was below the AI (73–92% of the AI) in most sex-age groups except in boys 7–10 years old. Inclusion of fortified foods and supplements increased median intakes in those sex-age groups closer to but still below the AI (88–94% of the AI). As such, no statement could be made on the adequacy of vitamin E. When excluding miss-reporters, median vitamin E intake from all sources was above the AI in almost all sex-age groups, except in children aged 4, 5 and 11–14 years. Therefore, the risk for inadequate vitamin E intake could be stated to be low. In those groups were median intake was below the AI, differences were minor. Considering the fact that an AI is higher than an eventual population reference intake (intake level adequate for 97.5% of the population) when possible to establish, it could be concluded that the risk for inadequate intakes of vitamin E in the Belgian population was low (Supplementary Table S8 and S9).

The risk for excessive intakes of vitamin E from the consumption of food, fortified food and supplements in the Belgian population was nil.

#### 4.4.4. Vitamin K

The mean usual vitamin K intake from food in the Belgian population was between 60.3 μg/day (girls 3–6 years) and 130.6 μg/day (men 40–64 years) (Table 9 and Table 10). Food was the main source for vitamin K. Fortified foods and supplements hardly contributed to total vitamin K intake. Intake data are presented for foods only, since aggregation with intake from fortified foods and supplements gave intake values that were, in some cases, lower than from foods only. This is due to the modelling effect and the low number of positive intakes from fortified foods and supplements.

The median vitamin K intake from food and from the aggregated consumption of food, fortified food and supplements was above the AI in all sex-age groups. The risk for inadequate intake of vitamin K in the Belgian population was therefore considered to be low. Except in women aged 15–17 and for some ages of adolescent men (15–17) and adults 18–39 (ic: inconclusive within a certain age-group), the median intake was below the AI (95–99% of the AI). Nevertheless, as the difference with the AI is minor and considering the nature of an AI, we consider the risk for inadequate intake of vitamin K to be low for the entire population. Total vitamin K intake from the aggregated consumption of all sources was below the UL. As a consequence, there was no risk for excessive intakes of vitamin K in the Belgium population.

Excluding miss-reporters (Supplementary Table S10 and S11) decreased the median intake in most sex-age groups. However, the conclusions within age groups remained the same.

## 5. Discussion

To our knowledge, the current study is the first evaluation of the risk of inadequate and excessive intakes of vitamins A, D, E and K in the general Belgian population taking account all sources, i.e., foods, fortified foods and supplements. Additionally, this study differentiates between mandatorily and voluntarily fortified foods as such, providing better insights in the current Belgian fortification and supplementation practices.

Our study revealed considerable inadequate intakes for vitamin A, from all sources, in the entire Belgian population. Median vitamin D intake from all sources was less than one third of the AI in all subgroups. The lowest intake values for vitamin A and D were found in adolescents. The risk for inadequate intake from vitamin E and K was low.

In Belgium, mandatory fortification of margarines and spreadable fats slightly decreased vitamin A inadequacies, but did not improve suboptimal intakes of vitamin D. Voluntarily fortified foods and supplements made a substantial contribution to the usual intake of vitamins D and E. Nonetheless, the effect on inadequacy risk for vitamin D was minor while inadequacies of vitamin E were substantially reduced. A small proportion of young children were at risk of exceeding the upper intake level of vitamin A, which was mainly related to higher intakes from the base diet. A minor proportion of adult woman and young children exceeded the UL for vitamin D when including supplements.

### 5.1. Intake and Inadequacies

Except for vitamin D, which is mainly produced under the influence of UVB-radiation, a well-balanced and healthy diet should provide the required amount of vitamins [2]. Inadequate vitamin A intake in the Belgium population could consequently be related to the unbalanced consumption of vegetables and dairy products, which are important food sources for vitamin A [35]. The consumption of vegetables and dairy products in 2014 was far below the reference values for the entire Belgian population, in particular in adolescents [41].

Because of the magnitude of the difference between median intake and the AI, we presume inadequate intake of vitamin D in all sex-age groups of the Belgian population. It should be kept in mind that these findings are valid under minimal sun exposure. Although, since Belgian life style is characterized by longer working days indoors and less outdoor activities, limited vitamin D body reserves from sun exposure might be more frequently occurring in Belgium than expected. Especially in winter-time when we rely on those body reserves, this might lead to inadequate status of vitamin D [5]. To our knowledge, no representative country data are available on vitamin D status in Belgium. The available studies are restricted to subgroups of the population and not always related to seasonal variability. However, an indication of the prevalence of low vitamin D status in Belgium is given by the studies of Sioen et al., Hoge et al. and Mac Farlane et al. that described high prevalence of vitamin D deficiency (25(OH)D <50 nmol/l) among Belgian children and adults [42,43,44].

Comparing dietary intake data with other European studies is hampered by the heterogeneity in assessment methods and by the difference in age classifications and in dietary reference values used to evaluate adequacy. Nevertheless, the present results are in line with European review studies when comparing intake levels. Higher intakes of vitamin A from foods are found in countries (Germany, Poland, Italy) with high consumption of sausages, liver and vegetables [3]. Higher vitamin D intake levels from food are found in Scandinavian countries were oil-rich fish and milk are more frequently consumed [4]. Our results for vitamin D intake from foods and fortified foods in young children (3–6 years) were somewhat higher, but in the same order as the results of the Flemish preschoolers [18].

Comparable to our study, notable discrepancies between mean intake and dietary recommendations for vitamins A and D are common in different European populations and in all sex-age groups. However, the proportion of inadequate vitamin A intakes in the Flemish preschoolers (4–10) were almost twice as high as in our study [18]. This can be explained by the higher reference values used in the latter study.

In contradiction to our results, Mensink et al. reported substantial proportions of inadequacies for vitamin E in all sex-age groups. However, intake data of fortified foods were generally lacking in this study [4]. Vitamin K intake data should be evaluated with caution due to uncertainties and limitation of food composition tables. Belgian intake data for vitamin K fell within the ranges reported in other European studies [36].

### 5.2. Fortified Foods and Supplements

The market of fortified foods and supplements in Europe is known to be a challenging market environment [10]. Despite a uniform European regulation on the addition of vitamins and minerals to foods (1925/2006), additional national legislations are adopted defining limited amounts of the added nutrients or national policies are set on mandatory fortification. As a result, fortification practices and consumption of fortified foods differ greatly between European countries [1]. In Belgium, children and adolescents generally consume more fortified foods than adults [40]. This reflects the substantial amount of foods designed for children and adolescents found in our inventory. The age-related decline of fortified food consumption is also substantiated by different European studies [1,12].

Although studies including the intake from fortified foods are limited, the available evidence indicates that voluntarily fortified foods can make a substantial contribution to dietary intakes and can reduce the risk of sub-optimal intakes at the population level of a range of micronutrients, among which are vitamins D and E [1]. Our results for vitamin E are in line with German results reporting fortified foods to be effective in raising low intake values of vitamin E closer to the reference values [1]. For vitamin D, however, we did not observe the same effect. Indeed, inclusion of fortified foods barely increased median vitamin D levels in the Belgian population.

In Europe, large differences exist in supplement consumption. Important contributions of supplements to total intake were frequently reported for vitamin D. Nevertheless, due to low uptake, supplements are often not effective as a public health strategy [12]. Our results are in line with these findings.

### 5.3. Excessive Intakes

In Europe, the risk for excessive intake of micronutrients taking into account all sources is low. Excessive intakes are mainly found in children, but the UL is exceeded by only a small proportion of children [1]. Concerning fat-soluble vitamins, excessive intakes were reported for retinol. Excessive intakes for retinol were mainly related to higher intakes from the base diet (Poland) or from supplements (Ireland). Fortified foods had little effect on the higher intakes (P95) due to the limited amounts of foods fortified with retinol. These results are in line with our findings.

The review studies of Hennessey and Flynn concluded that the risk of adverse health effects in individuals exceeding the UL was minor due to the safety margins used in establishing the UL and given the fact that the UL is exceeded by a modest amount [1,3]. In our study, the risk for high exposure of retinol is also limited in time, as it was only observed among the youngest age group.

In Europe, the risk for exceeding the UL of vitamin D is low, even in countries with a wide use of supplements [3,5]. In our study, high supplement intakes were generated from highly-dosed substances. In Belgium, vitamins with daily doses higher than 50 μg/day are considered as drugs. These drugs however are available at the pharmacist without prescription and therefore also taken into account in the usual intake estimations.

### 5.4. Strengths and Limitations

The importance of the evaluation of fortification and supplementation practices has been repeatedly stressed in previous works. The need to adapt survey methodologies to include intake data from all sources was hereby highlighted. Studies evaluating intake from all sources in Europe are scarce. These studies have been hampered by the complex research task required. Food and supplement composition tables need to be in line with the supply and nutritive values of fortified products on the market today [1,3,4]. To facilitate this, automatic adaptation of food composition tables with labelled values of fortified nutrient contents through bar-codes (e.g., Global standard one (GS1)) would encompass a serious reduction of the comprehensive task required.

An important strength of this study is that the contribution of fortified foods and supplements to total vitamin intake was evaluated. The survey methodology comprised a market inventory to assure that computation of vitamin intake was based on the most recent nutritive values. Furthermore, data were collected through the combination of two 24-h recalls and a supplementary FFQ, which is recommended by EFSA as a dietary assessment method [20,45]. Finally, vitamin intake was modelled with SPADE, a statistical program coping with multimodal distributions and heterogeneous variances between sources, typical for intake estimations from different sources [15,38].

This study however was confined to subjects aged 3–64 years. Besides the fortified foods presented in Table 1, our market investigation revealed a considerable amount of foods designed for infants and toddlers, like infant milk, follow-up milk, growing-up milk, milk cereals and dairy desserts for babies. The contribution of fortified foods and supplements might consequently differ greatly in these youngest age groups. This will have an impact on the risk of inadequate and excessive intake in those population subgroups and consequently on the national recommendations to be made in nutrition policy. It would therefore be advisable to additionally conduct ad hoc surveys in specific target populations like infants and toddlers.

A limitation of the study is that no biological samples were collected. For vitamin D, for which cutaneous synthesis excited by sun exposure is the main source, serum 25(OH)D levels would be required to give a picture on vitamin D status in the Belgian population and stress the magnitude of the evaluated vitamin D deficiency.

Another limitation of the study was that subjects with missing data of FFQ on supplements were all treated as non-users when they did not consume supplements on both the 24-h recalls. This conservative approach implies a possible underestimation of the intakes from supplements.

At last, the study did not include frequency questions on the consumption of dietary supplements. As a consequence, the intake values of subjects taking higher doses of supplements on a weekly or monthly basis had to be treated as users with missing data on the 24-h recall to enable modelling. This implies also a bias in the estimation of supplement intakes.

### 5.5. Recommendations

It is clear that fortification and supplementation practices in Belgium are insufficient to overcome inadequacies of vitamins A and D in the Belgium population. There is a need for dietary strategies to increase vitamin A and D intakes in the entire Belgian population. Intake data of the Belgian population provide an ideal starting point for modelling of fortification and supplementation scenarios. These scenario analyses will be very useful from a policy point of view. They should allow identifying which measures will ensure effective and safe increases in vitamin A and D intake. Fortification of margarines and fats in Belgium with vitamins A and D is currently based on nutritive values of butter. These scenario studies might give an insight into how a potential change in policy may affect intake levels of vitamins A and D at a population level [15,45,46]. However, it should be stressed that there is a need for nationally-representative data on vitamin D status in the Belgian population prior to setting out the final recommendations on vitamin D intake.

## 6. Conclusions

We present the first evaluation of the risk of inadequate and excessive intakes of vitamins A, D, E and K in the Belgian population taking into account foods, fortified foods and supplements. Belgian fortification and supplementation practices were insufficient to eradicate inadequate intakes of vitamins A and D. Excessive intakes were observed for vitamin A in a small proportion of children and for vitamin D in a small proportion of adult women and young children, although the risks associated with these excessive intakes were considered to be minor. By providing the first-ever dietary intake data of fat-soluble vitamins in Belgium from all sources, our study can support the development of adapted dietary strategies to increase vitamin A and D intakes in the general Belgian population while preventing excessive intakes.

## Figures and Tables

**Table 1:** Overview of the inventory of foods fortified with vitamins A, D, E and/or K and corresponding fortified value, suitable for the target population, Belgian study on the intake of vitamin A, D, E and K (VITADEK-study) 2015.

	Vitamin A		Vitamin D		Vitamin E		Vitamin K	
	Food Item	(μg/100 g)	Food Item	(μg/100 g)	Food Item	(mg/100 g)	Food Item	(μg/100 g)
Voluntarily-fortified foods	Fortified milk	120	Fortified milk	0.75–1	Fortified milk	1.8–2.4	Fortified milk	11.5
	Growing-up milk	63–87	Growing-up milk	1.1–3.1	Growing-up milk	0.83–1.1	Dairy drinks, probiotic drinks	11.5
	Dairy drinks, probiotic drinks	120	Dairy drinks, probiotic drinks	0.75–3.1	Milk substitutes	1.4–1.8	Growing-up milk	4–5.5
	Spreadable cheese	180	Milk substitutes	0.75–1.8	Margarines and spreadable fats	9.1–34	Fruit juices and lemonades	2
	Cacao powder	800–1263	Dairy desserts and substitutes	0.75–3.1	Cereals	10		
	Fruit juices and lemonades	100–400 ^(1)^	Cereals	2.9–7.5	Biscuits	3.6–7.2		
	Weight-loss products	233–1000	Biscuits	1.3–5.0	Fruit juices and lemonades	0.9–6		
			Fruit juices and lemonades	0.38	Cacao powder	12–15.8		
			Cacao powder	7.9–11	Weight-loss products	3.3–19		
			Weight-loss products	1.7–6.6				
Mandatorily-fortified foods	Margarines and spreadable fats	700–900	Margarines and spreadable fats	6–7.5				

^(1)^ Fortification under the form of provitamin A, i.e., β-carotene.

**Table 2:** Overview of the 5 most contributing food groups and the contribution of supplements to total vitamin A, D, E and K intake in the Belgian population 2014, Belgian food consumption survey 2014.

Vitamin A		Vitamin D		Vitamin E		Vitamin K	
Food Group	Contribution (%)	Food Group	Contribution (%)	Food Group	Contribution (%)	Food Group	Contribution (%)
Vegetables	31	Meat and meat products	27	Fat and oils	13	Vegetables	54
Dairy products	22	Dairy products	20	Vegetables	13	Dairy products	16
Fat and oils	13	Fat and oils	19	Sauces, spices, herbs and condiments	11	Fruits and nuts	11
Meat and meat products	8.0	Fish and shellfish	8.5	Meat and meat products	9.1	Fat and oils	9.0
Cakes and sweet biscuits	6.4	Cakes and sweet biscuits	7.4	Cakes and sweet biscuits	8.4	Sauces, spices, herbs and condiments	3.8
Supplements	2.5	Supplements	5.8	Supplements	3.5	Supplements	0.9

**Table 3:** Usual intake of vitamin A (µg/day) from food, fortified food and supplements in Belgian men (3–64 years), Belgian study on the intake of vitamins A, D, E and K (VITADEK-study) 2015.

				Percentiles							
Age		Usual Intake (CI)	% Contribution	5	50	95	97.5	EAR	% <EAR	UL	% >UL
3–6	Food	673 (615–762)	89.3	227	574	1452	1734	205–245 ^(1)^	6	800–1100 ^(1)^	2
	+ mandatorily fortified food	706 (657–783)	4.4	257	613	1468	1736		3		3
	+ voluntarily fortified food	744 (679–805)	5.0	277	646	1560	1827		2		3
	+ supplements	754 (698–831)	1.3	285	659	1554	1841		2		4
7–10	Food	693 (647–766)	90.7	233	591	1494	1784	320	14	1500	0
	+ mandatorily fortified food	732 (689–792)	5.1	271	631	1540	1813		9		0
	+ voluntarily fortified food	757 (706–809)	3.3	282	652	1572	1847		8		0
	+ supplements	764 (721–831)	0.9	289	669	1578	1851		7		1
11–14	Food	713 (667–774)	89.9	240	608	1537	1835	580	47	2000	0
	+ mandatorily fortified food	748 (712–809)	4.4	277	646	1578	1839		41		0
	+ voluntarily fortified food	771 (725–826)	2.9	289	667	1593	1929		40		0
	+ supplements	793 (739–844)	2.8	293	687	1656	1933		37		0
15–17	Food	731 (681–788)	90.1	246	624	1576	1882	570	44	2600	0
	+ mandatorily fortified food	775 (728–827)	5.4	287	664	1651	1948		38		0
	+ voluntarily fortified food	786 (740–845)	1.4	289	675	1635	1937		37		0
	+ supplements	811 (761–870)	3.1	304	702	1679	2005		34		0
18–39	Food	803 (728–856)	90.4	270	685	1735	2074	570	37	3000	0
	+ mandatorily fortified food	851 (774–908)	5.4	314	736	1776	2101		31		0
	+ voluntarily fortified food	862 (785–923)	1.2	320	748	1798	2115		30		0
	+ supplements	888 (809–937)	2.9	327	771	1844	2183		28		0
40–64	Food	937 (850–1033)	88.0	315	799	2026	2422	570	28	3000	0
	+ mandatorily fortified food	1050 (952–1143)	10.6	400	914	2154	2553		18		0
	+ voluntarily fortified food	1053 (960–1151)	0.3	403	919	2155	2542		17		0
	+ supplements	1065 (975–1163)	1.1	409	931	2172	2565		17		0

^(1)^ The EAR for vitamin A is 205 μg/day in children aged 1–3 years and 245 μg/day in children aged 4–6 years old. The UL for vitamin A (retinol) is 800 μg day in children aged 1–3 years and 1100 μg/day in children aged 4–6 years old. EAR: estimated average requirement; UL: upper intake level.

**Table 4:** Usual intake of vitamin A (µg/day) from food, fortified food and supplements in Belgian women (3–64 years), Belgian study on the intake of vitamins A, D, E and K (VITADEK-study) 2015.

				Percentiles							
Age		Usual Intake (CI)	% Contribution	5	50	95	97.5	EAR	% <EAR	UL	% >UL
3–6	Food	606 (563–649)	86.4	238	540	1196	1388	205–245 ^(1)^	5	800–1000 ^(1)^	0
	+ mandatorily fortified food	659 (612–696)	7.6	273	594	1275	1468		3		0
	+ voluntarily fortified food	671 (635–718)	1.7	290	608	1261	1461		2		0
	+ supplements	701 (645–747)	4.3	299	636	1316	1521		1		1
7–10	Food	621 (585–659)	87.3	244	554	1225	1422	320	13	1500	0
	+ mandatorily fortified food	667 (633–705)	6.5	278	599	1282	1488		9		0
	+ voluntarily fortified food	693 (651–723)	3.7	297	629	1335	1548		7		0
	+ supplements	711 (663–740)	2.5	306	648	1356	1534		6		0
11–14	Food	636 (600–673)	88.2	250	568	1255	1456	490	38	2000	0
	+ mandatorily fortified food	682 (646–719)	6.4	284	615	1316	1521		31		0
	+ voluntarily fortified food	698 (663–734)	2.2	298	628	1337	1528		29		0
	+ supplements	721 (675–753)	3.2	311	653	1352	1567		26		0
15–17	Food	650 (613–687)	89.5	256	580	1281	1487	490	37	2600	0
	+ mandatorily fortified food	694 (657–734)	6.1	295	626	1308	1503		30		0
	+ voluntarily fortified food	709 (673–746)	2.1	296	637	1364	1578		28		0
	+ supplements	726 (688–770)	2.3	311	659	1358	1544		26		0
18–39	Food	705 (654–754)	82.6	277	629	1388	1612	490	31	3000	0
	+ mandatorily fortified food	749 (701–797)	5.2	319	675	1429	1649		24		0
	+ voluntarily fortified food	768 (723–816)	2.2	327	693	1462	1690		22		0
	+ supplements	853 (790–931)	10.0	349	753	1698	2006		18		0
40–64	Food	805 (740–875)	86.6	319	719	1582	1833	490	22	3000	0
	+ mandatorily fortified food	868 (803–947)	6.8	370	784	1655	1898		15		0
	+ voluntarily fortified food	881 (810–958)	1.4	379	796	1669	1930		14		0
	+ supplements	930 (848–1014)	5.3	404	845	1742	1988		11		0

^(1)^ EAR for vitamin A is 205 μg/day in children aged 1–3 years and 245 μg/day in children aged 4–6 years old. The UL for vitamin A (retinol) is 800 μg day in children aged 1–3 years and 1100 μg/day in children aged 4–6 years old. EAR: estimated average requirement; UL: upper intake level.

**Table 5:** Usual intake of vitamin D (µg/day) from food, fortified food and supplements in Belgian men (3–64 years), Belgian study on the intake of vitamins A, D, E and K (VITADEK-study) 2015. AI, adequate intake.

				Percentiles							
Age		Usual Intake (CI)	% Contribution	5	50	95	97.5	AI	% Inadequate ^(1)^	UL	% >UL
3–6	Food	2.34 (2.08–2.95)	20.2	0.72	2.01	5.11	6.06	15	ns ^(2)^	50	0.0
	+ mandatorily fortified food	2.71 (2.4–3.31)	3.2	0.99	2.39	5.48	6.49	15	ns		0.0
	+ voluntarily fortified food	3.69 (3.4–4.31)	8.4	1.44	3.37	7.01	7.99	15	ns		0.0
	+ supplements	11.61 (4.4–19.97)	68.2	1.56	3.89	13.91	51.76	15	ns		2.5
7–10	Food	2.82 (2.5–3.2)	53.4	0.90	2.44	6.06	7.16	15	ns	50	0.0
	+ mandatorily fortified food	3.20 (2.9–3.63)	7.2	1.18	2.82	6.58	7.63	15	ns		0.0
	+ voluntarily fortified food	3.98 (3.7–4.41)	14.8	1.56	3.58	7.80	8.89	15	ns		0.0
	+ supplements	5.28 (4.16–7.12)	24.6	1.61	3.84	9.09	11.26	15	ns		0.4
11–14	Food	3.07 (2.74–3.39)	63.3	0.99	2.65	6.55	7.72	15	ns	100	0.0
	+ mandatorily fortified food	3.46 (3.13–3.83)	8.0	1.30	3.05	7.01	8.00	15	ns		0.0
	+ voluntarily fortified food	4.04 (3.69–4.41)	12.0	1.57	3.64	7.84	9.04	15	ns		0.0
	+ supplements	4.85 (4.05–11.51)	16.7	1.66	3.81	8.92	10.81	15	ns		0.2
15–17	Food	3.21 (2.83–3.47)	60.9	1.04	2.78	6.83	8.05	15	ns	100	0.0
	+ mandatorily fortified food	3.65 (3.24–3.97)	8.3	1.31	3.19	7.45	8.01	15	ns		0.0
	+ voluntarily fortified food	4.08 (3.68–4.41)	8.2	1.57	3.65	8.04	9.39	15	ns		0.0
	+ supplements	5.27 (3.98–7.64)	22.6	1.66	3.85	9.15	11.21	15	ns		0.3
18–39	Food	3.49 (3.10–3.68)	56.7	1.14	3.02	7.39	8.70	15	ns	100	0.0
	+ mandatorily fortified food	3.99 (3.61–4.24)	8.1	1.5	3.54	8.01	9.28	15	ns		0.0
	+ voluntarily fortified food	4.34 (3.92–4.58)	5.7	1.68	3.89	8.47	9.82	15	ns		0.0
	+ supplements	6.15 (4.51–8.07)	29.4	1.73	4.09	9.86	12.26	15	ns		0.5
40–64	Food	3.71 (3.38–4.04)	51.6	1.23	3.23	7.85	9.23	15	ns	100	0.0
	+ mandatorily fortified food	4.71 (4.27–5.19)	13.9	1.90	4.24	9.14	10.56	15	ns		0.0
	+ voluntarily fortified food	4.88 (4.48–5.37)	2.4	1.99	4.41	9.34	10.76	15	ns		0.0
	+ supplements	7.19 (4.86–10.67)	32.1	2.05	4.61	10.68	13.58	15	ns		0.6

^(1)^ When not enough evidence is available to set an EAR, an AI is set. The AI is based on the average intake of an apparently healthy population. ^(2)^ When the median intake of the population > the AI, the risk for inadequate intake is low. No statement (ns) can be formulated on the adequacy of vitamin D when the median intake < AI. EAR: Estimated average requirement; UL: upper intake level; AI: adequate intake.

**Table 6:** Usual intake of vitamin D (µg/day) from food, fortified food and supplements in Belgian women (3–64 years), Belgian study on the intake of vitamins A, D, E and K (VITADEK-study) 2015.

				Percentiles							
Age		Usual Intake (CI)	% Contribution	5	50	95	97.5	AI	% Inadequate ^(1)^	UL	% >UL
3–6	Food	2.75 (2.32–3.02)	29.7	0.95	2.41	5.69	6.66	15	ns ^(2)^	25	0.0
	+ mandatorily fortified food	3.05 (2.65–3.33)	3.2	1.19	2.73	6.03	7.06	15	ns		0.0
	+ voluntarily fortified food	3.79 (3.39–4.11)	8.0	1.60	3.48	6.98	8.05	15	ns		0.0
	+ supplements	9.27 (4.49–18.1)	59.1	1.73	3.84	14.54	25.08	15	ns		1.7
7–10	Food	2.75 (2.47–3.03)	38.1	0.95	2.41	5.70	6.67	15	ns	25	0.0
	+ mandatorily fortified food	3.04 (2.79–3.33)	4.0	1.21	2.71	6.04	6.96	15	ns		0.0
	+ voluntarily fortified food	3.66 (3.39–3.95)	8.6	1.51	3.33	6.89	7.92	15	ns		0.0
	+ supplements	7.21 (4.54–11.5)	49.2	1.56	3.64	10.85	16.43	15	ns		1.1
11–14	Food	2.76 (2.49–3.03)	49.3	0.95	2.42	5.72	6.69	15	ns	50	0.0
	+ mandatorily fortified food	3.04 (2.81–3.33)	5.0	1.19	2.74	5.98	6.91	15	ns		0.0
	+ voluntarily fortified food	3.51 (3.28–3.83)	8.4	1.43	3.22	6.58	7.48	15	ns		0.0
	+ supplements	5.62 (3.81–7.4)	37.3	1.45	3.43	9.48	13.69	15	ns		0.5
15–17	Food	2.76 (2.51–3.04)	46.9	0.95	2.43	5.73	6.70	15	ns	50	0.0
	+ mandatorily fortified food	3.07 (2.83–3.35)	5.3	1.21	2.73	5.98	7.06	15	ns		0.0
	+ voluntarily fortified food	3.46 (3.21–3.80)	6.6	1.39	3.15	6.58	7.50	15	ns		0.0
	+ supplements	5.89 (3.71–7.0)	41.3	1.43	3.33	9.04	13.13	15	ns		0.5
18–39	Food	2.79 (2.54–3.07)	30.8	0.96	2.45	5.77	6.75	15	ns	50	0.0
	+ mandatorily fortified food	3.20 (2.95–3.47)	4.5	1.27	2.87	6.24	7.21	15	ns		0.0
	+ voluntarily fortified food	3.51 (3.26–3.85)	3.4	1.42	3.18	6.71	7.67	15	ns		0.0
	+ supplements	9.07 (4.88–13.4)	61.3	1.55	3.64	14.39	26.23	15	ns		1.4
40–64	Food	2.82 (2.54–3.12)	21.5	0.98	2.48	5.84	6.84	15	ns	50	0.0
	+ mandatorily fortified food	3.45 (3.11–3.77)	4.8	1.40	3.12	6.63	7.64	15	ns		0.0
	+ voluntarily fortified food	3.80 (3.48–4.17)	2.7	1.59	3.47	7.09	8.16	15	ns		0.0
	+ supplements	13.15 (6.09–24.1)	71.0	1.77	4.17	22.59	44.0	15	ns		1.9

^(1)^ When not enough evidence is available to set an EAR, an AI is set. The AI is based on the average intake of an apparently healthy population. ^(2)^ When the median intake of the population >the AI, the risk for inadequate intake is low. No statement (ns) can be formulated on the adequacy of vitamin D when the median intake <AI. EAR: estimated average requirement; UL: upper intake level; AI: adequate intake.

**Table 7:** Usual intake of vitamin E (mg/day) from food, fortified food and supplements in Belgian men (3–64 years), Belgian study on the intake of vitamins A, D, E and K (VITADEK-study) 2015.

				Percentiles							
Age		Usual Intake (CI)	% Contribution	5	50	95	97.5	AI ^(2)^	% Inadequate ^(2)^	UL	% >UL
3–6	Food	7.6 (7.1–8.1)	82.6	3.4	7.0	14.0	16.0	6–9 ^(1)^	Ns ^(3)^	60	0
	+ fortified food	9.0 (8.3–9.3)	15.2	4.1	8.3	16.2	18.2		Ic ^(4)^		0
	+ supplements	9.2 (8.6–9.6)	2.2	4.2	8.5	16.7	18.8		ic		0
7–10	Food	9.9 (9.4–10.6)	88.4	4.6	9.2	18.0	20.4	9	Low ^(3)^	100	0
	+ fortified food	10.8 (10.3–11.4)	8.0	5.1	10.0	19.0	21.7		low		0
	+ supplements	11.2 (10.6–11.7)	3.6	5.1	10.3	20.0	22.6		low		0
11–14	Food	11.4 (10.76–12.1)	91.9	5.3	10.5	20.5	23.2	13	ns	120	0
	+ fortified food	12.1 (11.5–12.7)	5.6	5.8	11.3	21.4	24.5		ns		0
	+ supplements	12.4 (11.8–13.0)	2.4	5.9	11.6	22.2	25.0		ns		0
15–17	Food	12.2 (11.54–12.8)	92.4	5.7	11.3	21.9	24.8	13	ns	130	0
	+ fortified food	12.9 (12.2–13.4)	5.3	6.1	11.9	22.9	25.7		ns		0
	+ supplements	13.2 (12.5–13.8)	2.3	6.2	12.3	23.4	26.7		ns		0
18–39	Food	13.0 (12.17–13.6)	92.2	6.1	12.0	23.3	26.4	13	ns	150	0
	+ fortified food	13.6 (12.8–14.3)	4.3	6.4	12.6	24.1	27.2		ic		0
	+ supplements	14.1 (13.2–14.8)	3.5	6.6	13.0	25.0	28.3		ic		0
40–64	Food	11.7 (11.01–12.4)	87.3	5.4	10.8	21.1	24.0	13	ns	150	0
	+ fortified food	12.8 (11.9–13.5)	8.2	6.0	11.8	22.7	25.5		ic		0
	+ supplements	13.4 (12.6–14.3)	4.5	6.2	12.2	24.4	28.2		ic		0

^(1)^ The AI for vitamin E is 6 mg/day in children aged 1–3 years and 9 mg/day in children aged 4–6 years old. ^(2)^ When not enough evidence is available to set an EAR, an AI is set. The AI is based on the average intake of an apparently healthy population. ^(3)^ When the median intake of the population lies above the AI, the risk for inadequate intake is low. When the median intake <AI, no statement (ns) can be formulated on the the adequacy of vitamin E. ^(4)^ ic: inconclusive within a certain age-group. EAR: estimated average requirement; UL: upper intake level; AI: adequate intake.

**Table 8:** Usual intake of vitamin E (mg/day) from food, fortified food and supplements in Belgian women (3–64 years), Belgian study on the intake of vitamins A, D, E and K (VITADEK-study) 2015.

				Percentiles							
Age		Usual Intake (CI)	% Contribution	5	50	95	97.5	AI ^(2)^	% Inadequate ^(3)^	UL	% >UL
3–6	Food	7.0 (6.6–7.6)	82.4	3.4	6.6	12.2	13.7	6–9 ^(1)^	ns	60	0
	+ fortified food	8.0 (7.5–8.5)	11.8	3.9	7.5	13.9	15.7		ic ^(4)^		0
	+ supplements	8.5 (8.0–9.1)	5.9	4.1	7.9	15.3	17.6		ic		0
7–10	Food	8.8 (8.4–9.3)	87.1	4.5	8.3	14.9	16.6	9	ns	100	0
	+ fortified food	9.7 (9.2–10.1)	8.9	4.9	9.1	16.4	18.1		ic		0
	+ supplements	10.1 (9.5–10.5)	4	5.0	9.5	17.3	19.4		low		0
11–14	Food	9.5 (8.8–9.9)	89.6	4.9	9.0	16.0	17.8	11	ns	120	0
	+ fortified food	10.3 (9.7–10.7)	7.5	5.3	9.7	7.2	19.2		ns		0
	+ supplements	10.6 (9.9–11.0)	2.8	5.3	9.9	18.1	20.3		ns		0
15–17	Food	9.8 (9.0–10.1)	90.7	5.0	9.2	16.4	18.2	11	ns	130	0
	+ fortified food	10.5 (9.8–10.9)	6.5	5.5	9.9	17.5	19.4		ns		0
	+ supplements	10.8 (10.1–11.3)	2.8	5.5	10.2	18.3	20.6		ns		0
18–39	Food	9.7 (9.1–10.1)	83.6	5.0	9.1	16.2	18.1	11	ns	150	0
	+ fortified food	10.4 (9.9–11.0)	6	5.4	9.8	17.4	19.3		ns		0
	+ supplements	11.6 (10.9–13.1)	10.3	5.6	10.5	20.4	23.6		ns		0
40–64	Food	8.9 (8.5–9.6)	80.2	4.5	8.4	15.0	16.7	11	ns	150	0
	+ fortified food	9.5 (9.0–10.4)	5.4	4.9	9.0	16.0	17.8		ns		0
	+ supplements	11.1 (10.3–12.0)	14.4	5.1	9.8	21.1	25.3		ns		0

^(1)^ AI for vitamin E is 6 mg/day in children aged 1-3 years and 9 mg/day in children aged 4–6 years old. ^(2)^ When not enough evidence is available to set an EAR, an AI is set. The AI is based on the average intake of an apparently healthy population. ^(3)^ When the median intake of the population lies above the AI, the risk for inadequate intake is low. Since the distribution of requirements is not known, no statement (ns) can be formulated on the adequacy of vitamin E when the median intake is lower than the AI. ^(4)^ ic: inconclusive within a certain age-group. EAR: Estimated average requirement; UL: upper intake level; AI: adequate intake.

**Table 9:** Usual intake of vitamin K (µg/day) from foods only in Belgian men (3–64 years), Belgian study on the intake of vitamins A, D, E and K (VITADEK-study) 2015.

			Percentiles							
Age		Usual Intake (CI)	5	50	95	97.5	AI ^(2)^	% Inadequate ^(3)^	UL	% >UL
3–6	Food	64 (56–73)	21	54	140	168	12–20 ^(1)^	low	200–300 ^(1)^	0.5
7–10	Food	63 (57–68)	21	53	136	163	30	low	450	0.0
1–14	Food	69 (64–74)	23	58	150	180	45	low	750	0.0
15–17	Food	76 (70–82)	25.1	65	166	199	65	ic^(4)^	900	0.0
18–39	Food	111 (103–121)	35	93	249	301	70	ic	1000	0.0
40–64	Food	185 (172–202)	59	156	412	496	70	low	1000	0.1

^(1)^ AI for vitamin K is 12 μg/day in children aged 1–3 years and 20 μg/day in children aged 4–6 years old. The UL for vitamin K is 200 μg/day in children aged 1–3 years and 300 μg/day in children aged 4–6 years old. ^(2)^ When not enough evidence is available to set an EAR, an AI is set. The AI is based on the average intake of an apparently healthy population. ^(3)^ When the median intake of the population lies above the AI, the risk for inadequate intake is low. Since the distribution of requirements is not known, no statement (ns) can be formulated on the adequacy of vitamin K when the median intake is lower than the AI. ^(4)^ ic: inconclusive within a certain age-group. EAR: estimated average requirement; UL: upper intake level; AI: adequate intake.

**Table 10:** Usual intake of vitamin K (µg/day) from foods only in Belgian women (3–64 years), Belgian study on the intake of vitamins A, D, E and K (VITADEK-study) 2015.

			Percentiles							
Age		Usual Intake (CI)	5	50	95	97.5	AI ^(2)^	% Inadequate ^(3)^	UL	% >UL
3–6	Food	54 (47–61)	21	47	109	128	12–20 ^(1)^	low	200–300 ^(1)^	0.1
7–10	Food	58 (55–64)	22	51	118	139	30	low	450	0.0
11–14	Food	65 (61–71)	25	57	131	154	45	low	750	0.0
15–17	Food	71 (66–79)	27	63	145	170	65	ns	900	0.0
18–39	Food	108 (99–116)	38	94	228	270	70	ic^(4)^	1000	0.0
40–64	Food	176 (164–192)	66	154	360	424	70	low	1000	0.0

^(1)^ AI for vitamin K is 12 μg/day in children aged 1–3 years and 20 μg/day in children aged 4–6 years old. The UL for vitamin K is 200 μg/day in children aged 1–3 years and 300 μg/day in children aged 4–6 years old. ^(2)^ When not enough evidence is available to set an EAR, an AI is set. The AI is based on the average intake of an apparently healthy population. ^(3)^ When the median intake of the population lies above the AI, the risk for inadequate intake is low. Since the distribution of requirements is not known, no statement (ns) can be formulated on the adequacy of vitamin K when the median intake is lower than the AI. ^(4)^ ic: inconclusive within a certain age-group. EAR: estimated average requirement; UL: upper intake level; AI: adequate intake; ns: no statement.

## Supplementary Materials

The following are available online at www.mdpi.com/2072-6643/9/8/860/s1: Table S1: Contribution of food groups to total intake of vitamins A, D, E and K, Belgian population, 2014; Table S2: Usual intake of retinol (μg/day) from food, fortified food and supplements in Belgian men (3–64 years), according to age and gender, Belgian VITADEK-study 2015; Table S3: Usual intake of retinol (μg/day) from food, fortified food and supplements in Belgian women (3–64 years), according to age and gender, Belgium, 2014; Table S4: Usual intake of vitamin A (µg/dag) from food, fortified food and supplements in Belgian men (3–64 years), excluding miss-reporters, according to age and gender, Belgian VITADEK-study 2015; Table S5: Usual intake of vitamin A (µg/dag) from food, fortified food and supplements in Belgian women (3–64 years), excluding miss-reporters, according to age and gender, Belgian VITADEK-study 2015; Table S6: Usual intake of vitamin D (µg/day) from food, fortified food and supplements in Belgian men (3–64 years), excluding miss-reporters, according to age and gender, Belgian VITADEK-study 2015; Table S7: Usual intake of vitamin D (µg/day) from food, fortified food and supplements in Belgian women (3–64 years), excluding miss-reporters, according to age and gender, Belgian VITADEK-study 2015; Table S8: Usual intake of vitamin E (mg/day) from food, fortified food and supplements in Belgian men (3–64 years), excluding miss-reporters, according to age and gender, Belgian VITADEK-study 2015; Table S9: Usual intake of vitamin E (mg/day) from food, fortified food and supplements in Belgian women (3–64 years), excluding miss-reporters, according to age and gender, Belgian VITADEK-study 2015; Table S10: Usual intake of vitamin K (μg/day) from foods only in Belgian men (3–64 years), excluding miss-reporters, according to age and gender, Belgian VITADEK-study 2015; Table S11: Usual intake of vitamin K (μg/day) from foods only in Belgian women (3–64 years), excluding miss-reporters, according to age and gender, Belgian VITADEK-study 2015.

## References

[1] Hennessy A., Walton J., Flynn A. (2013). The impact of voluntary food fortification on micronutrient intakes and status in European countries: A review. Proc. Nutr. Soc..

[2] Verkaik-Kloosterman J., McCann M.T., Hoekstra J., Verhagen H. (2012). Vitamins and minerals: Issues associated with too low and too high population intakes. Food Nutr. Res..

[3] Flynn A., Hirvonen T., Mensink G.B., Ocké M.C., Serra-Majem L., Stos K., Szponar L., Tetens I., Turrini A., Fletcher R. (2009). Intake of selected nutrients from foods, from fortification and from supplements in various European countries. Food Nutr. Res..

[4] Mensink G.B., Fletcher R., Gurinovic M., Huybrechts I., Lafay L., Serra-Majem L., Szponar L., Tetens I., Verkaik-Kloosterman J., Baka A. (2013). Mapping low intake of micronutrients across Europe. Br. J. Nutr..

[5] Spiro A., Buttriss J.L. (2014). Vitamin D: An overview of vitamin D status and intake in Europe. Nutr. Bull..

[6] World Health Organization (2015). European Food and Nutrition Action Plan 2015–2020.

[7] Dwyer J.T., Wiemer K.L., Dary O., Keen C.L., King J.C., Miller K.B., Philbert M.A., Tarasuk V., Taylor C.L., Gaine P.C. (2015). Fortification and health: Challenges and opportunities. Adv. Nutr..

[8] Fulgoni V.L., Keast D.R., Bailey R.L., Dwyer J. (2011). Foods, fortificants, and supplements: Where do Americans get their nutrients?. J. Nutr..

[9] Berner L.A., Keast D.R., Bailey R.L., Dwyer J.T. (2014). Fortified foods are major contributors to nutrient intakes in diets of US children and adolescents. J. Acad. Nutr. Diet..

[10] Özen A.E., Bibiloni Mdel M., Pons A., Tur J.A. (2014). Consumption of functional foods in Europe, a systematic review. Nutr. Hosp..

[11] Hannon E.M., Kiely M., Flynn A. (2007). The impact of voluntary fortification of foods on micronutrient intakes in Irish adults. Br. J. Nutr..

[12] Hennessy A., Browne F., Kiely M., Walton J., Flynn A. (2017). The role of fortified foods and nutritional supplements in increasing vitamin D intake in Irish preschool children. Eur. J. Nutr..

[13] Laaksi I.T., Ruohola J.P., Ylikomi T.J., Auvinen A., Haataja R.I., Pihlajamäki H.K., Tuohimaa P.J. (2006). Vitamin D fortification as public health policy: Significant improvement in vitamin D status in young Finnish men. Eur. J. Clin. Nutr..

[14] European Food Safety Authority (2006). Tolerable upper Intake Levels for Vitamins and Minerals.

[15] Verkaik-Kloosterman J., Beukers M.H., Jansen-van der Vliet M., Ocké M.C. (2017). Vitamin D intake of Dutch infants from the combination of (fortified) foods, infant formula and dietary supplements. Eur. J. Nutr..

[16] Superior Health Council (2015). Voedingsaanbevelingen voor België–Herziening 2015–2016. Partim I: Vitaminen en Sporenelementen.

[17] Huybrechts I., De Henauw S. (2007). Energy and nutrient intakes by pre-school children in Flanders-Belgium. Br. J. Nutr..

[18] Bel S., Van den Abeele S., Lebacq T., Ost C., Brocatus L., Stiévenart C., Teppers E., Tafforeau J., Cuypers K. (2016). Protocol of the Belgian food consumption survey 2014: Objectives, design and methods. Arch. Public Health.

[19] European Food Safety Authority (2014). Guidance on the EU menu methodology. EFSA J..

[20] NUBEL Extraction of the Belgian Food Composition Database and Food Composition Brand Name Database (NIMS: Nubel Information Management system) 2015. http://www.internubel.be/.

[21] NEVO-online Versie 2016/5.0, ©RIVM. http://www.rivm.nl/nevo/.

[22] Bech-Larsen T., Scholderer J. (2007). Functional foods in Europe: Consumer research, market experiences and regulatory aspects. Trends Food Sci. Technol..

[23] Farma Compendium +. http://www.farmacompendium.be/farma/.

[24] Mc Cance and Widdowsons’ Composition of Foods Integrated Dataset 2016. https://www.gov.uk/government/publications/composition-of-foods-integrated-dataset-cofid.

[25] Table Ciqual 2016: French Food Composition Table. https://pro.anses.fr/TableCIQUAL/index.htm.

[26] (2016). Danish Food Composition Data Version 7.01. http://www.foodcomp.dk/v7/fcdb_namesearch.asp.

[27] National Nutrition Database for Standard Reference 2016. https://ndb.nal.usda.gov/ndb/.

[28] Bolton-Smith C., Price R., Fenton S., Harrington D., Shearer M. (2000). Compilation of a provisional UK database for the phylloquinone (vitamin K1) content of foods. Br. J. Nutr..

[29] Moyersoen I., Demarest S., De Ridder K., Tafforeau J., Lachat C., Van Camp J. (2017). Fat soluble vitamin intake from the consumption of food, fortified foods and supplements: Design and methods of the Belgian VITADEK study. Arch. Public Health.

[30] Cuypers K., Lebacq T., Bel S., Lebacq T., Teppers E. (2016). Inleiding en methode. Voedselconsumptiepeiling 2014–2015. Rapport 4.

[31] Dekkers A.L., Verkaik-Kloosterman J., van Rossum C.T., Ocké M.C. (2014). SPADE, a new statistical program to estimate habitual dietary intake from multiple food sources and dietary supplements. J. Nutr..

[32] European Food Safety Authority (2015). Scientific opinion on dietary reference values for vitamin E as α-tocopherol. EFSA J..

[33] European Food Safety Authority (2016). Scientific opinion on dietary reference values for vitamin D1. EFSA J..

[34] European Food Safety Authority (2015). Scientific opinion on dietary reference values for vitamin A. EFSA J..

[35] Turck D., Bresson J.-L., Burlingame B., Dean T., Fairweather-Tait S., Heinonen M., Hirsch-Ernst K.I., Mangelsdorf I., McArdle H.J., EFSA NDA Panel (EFSA Panel on Dietetic Products, Nutrition and Allergies) (2017). Dietary reference values for vitamin K. EFSA J..

[36] EFSA Panel on Dietetic Products, Nutrition, and Allergies (NDA) (2010). Scientific opinion on principles for deriving and applying dietary reference values. EFSA J..

[37] Dekkers A., Verkaik-Kloosterman J., Van Rossum C., Ocké M.C. (2014). SPADE: Statistical Program to Assess habitual Dietary Exposure. User's Manual. Version 2.0.

[38] Block G., Hartman A.M., Dresser C.M., Carroll M.D., Gannon J., Gardner L. (1986). A data-based approach to diet questionnaire design and testing. Am. J. Epidemiol..

[39] De Ridder K., Bel S., Tafforeau J. (2016). Energie. Voedselconsumptiepeiling 2014–2015. Rapport 4.

[40] De Ridder K., Bel S., Brocatus L., Lebacq T., Ost C., Teppers E., Tafforeau J. (2016). Samenvatting van de resultaten. Voedselconsumptiepeiling 2014–2015.

[41] Sioen I., Moratidou T., Kaufman J., Bammann K., Michels N., Vanaelst B., Vyncke K., De Henauw S. (2011). Vitamin D status of Belgian young children. 1st IOF-ESCEO Pre-Clinical Symposium..

[42] Hoge A., Donneau A., Streel S., Kohl P., Chapelle J., Albert A., Cavalier E., Guillaume M. (2015). Vitamin D deficiency is common among adults in Wallonia (Belgium, 51°30′ North): Findings from the Nutrition, Environment and Cardio-Vascular Health study. Nutr. Res..

[43] MacFarlane G., Sackrison J., Body J., Ersfeld D., Fenske J., Miller A. (2004). Hypovitaminosis D in a normal, apparently healthy urban European population. J. Steroid Biochem..

[44] Carroll R.J., Midthune D., Subar A.F., Shumakovich M., Freedman L.S., Thompson F.E., Kipnis V. (2012). Taking advantage of the strengths of 2 different dietary assessment instruments to improve intake estimates for nutritional epidemiology. Am. J. Epidemiol..

[45] Raulio S., Erlund I., Männnistö S., Sarlio-Lähteenkorva S., Sundvall J., Tapanainen H., Vartiainen E., Virtanen S.M. (2017). Successful nutrition policy: Improvement of vitamin D intake and status in Finnish adults over the last decade. Eur. J. Public Health.

[46] Harika R., Dötsch-Klerk M., Zock P., Eilander A. (2016). Compliance with dietary guidelines and increased fortification can double vitamin D intake: a simulation study. Ann. Nutr. Metab..

