# Supplementary material for: Intake of Fat-Soluble Vitamins in the Belgian Population: Adequacy and Contribution of Foods, Fortified Foods and Supplements
Source: Nutrients. 2017 Aug 11;9(8):860. doi: 10.3390/nu9080860 (PMC5579653; doi:10.3390/nu9080860)
Supplement: Supplementary file 1 [file nutrients-09-00860-s001.pdf]

Table S1. Contribution of food groups to total intake of vitamin A, D, E and K, Belgian population, Belgian food consumption survey 2014

FOOD GROUPS	Mean contribution to total vitamin A intake in 2014 (%)	Mean contribution to total vitamin D intake in 2014 (%)	Mean contribution to total vitamin E intake in 2014 (%)	Mean contribution to total vitamin K intake in 2014 (%)
Potatoes and other tubers	0.2 (0.2-0.3)	0.0 (0.0-0.0)	2.5 (2.2-2.8)	0.3 (0.0-0.7)
Vegetables	31.4 (29.9-33.0)	0.0 (0.0-0.0)	13.1 (12.2-13.9)	53.7 (51.8-55.7)
Legumes	0.0 (0.0-0.0)	0.0 (0.0-0.0)	0.1 (0.1-0.2)	0.7 (0.4-1.0)
Fruits, nuts and seeds	2.6 (2.3-2.9)	0.0 (0.0-0.0)	7.1 (6.4-7.7)	10.8 (9.7-11.9)
Dairy products	22.0 (20.9-23.2)	20.4 (19.1-21.8)	5.7 (5.2-6.1)	16.2 (14.8-17.6)
Cereals and cereal products	0.4 (0.3-0.4)	2.6 (2.1-3.1)	5.7 (5.2-6.1)	1.3 (1.0-1.6)
Meat and meat products	8.0 (7.0-9.1)	27.3 (24.9-29.8)	9.1 (8.3-9.8)	0.1 (0.0-0.2)
Fish and shellfish	0.8 (0.5-1.0)	8.5 (7.3-9.7)	3.6 (3.0-4.2)	0.0 (0.0-0.0)
Eggs and egg products	2.8 (2.4-3.2)	5.0 (4.3-5.8)	3.4 (2.9-3.9)	0.0 (0.0-0.1)
Fat	13.4 (12.5-14.2)	18.5 (17.1-19.8)	13.1 (12.2-14.1)	9.1 (7.9-10.2)
Sugar and confectionery	1.7 (1.4-2.0)	3.3 (2.5-4.0)	7.7 (6.8-8.5)	0.0 (0.0-0.0)
Cakes and sweet biscuits	6.4 (5.7-7.1)	7.4 (6.6-8.2)	8.4 (7.7-9.2)	2.8 (2.1-3.4)
Non-alcoholic beverages	2.6 (1.9-3.2)	0.1 (0.0-0.1)	3.1 (2.5-3.7)	0.0 (0.0-0.1)
Alcoholic beverages	0.0 (0.0-0.0)	0.0 (0.0-0.0)	0.0 (0.0-0.0)	0.0 (0.0-0.0)
Sauces, spices, herbs and condiments	4.5 (4.0-5.0)	2.5 (2.0-3.0)	11.4 (10.4-12.4)	3.8 (3.1-4.5)
Soups, bouillon	0.0 (0.0-0.0)	0.0 (0.0-0.0)	0.0 (0.0-0.0)	0.0 (0.0-0.0)
Miscellaneous*	0.4 (0.2-0.7)	0.4 (0.2-0.6)	0.3 (0.2-0.5)	0.0 (0.0-0.0)
Savoury snacks**	0.3 (0.2-0.5)	0.3 (0.2-0.5)	2.2 (1.9-2.6)	0.2 (0.0-0.4)
Food supplements	2.5 (1.8-3.1)	5.8 (4.7-6.9)	3.5 (2.8-4.3)	0.9 (0.6-1.2)

Table S2. Usual intake of retinol (µg/day) from food, fortified food and supplements in Belgian men (3-64 years), Belgian study on the intake of vitamins A, D, E and K (VITADEK-study) 2015

Gender/age		Usual intake	Percentiles 5	50	95	97.5	UL	% > UL
**MEN**								
**3-6**	Food	338	96	280	773	932	800-1100 (1)	2
	+ mandatorily fortified food	378	125	324	811	979	800	3
	+ voluntarily fortified food	397	132	339	869	1024	800	3
	+ supplements	410	135	350	886	1069	800	4
**7-10**	Food	339	96	282	776	937	1500	0
	+ mandatorily fortified food	383	128	327	843	988	1500	0
	+ voluntarily fortified food	399	130	336	866	1036	1500	0
	+ supplements	411	139	352	886	1047	1500	0
**11-14**	Food	342	97	284	782	944	2000	0
	+ mandatorily fortified food	385	132	329	838	975	2000	0
	+ voluntarily fortified food	396	136	337	854	1023	2000	0
	+ supplements	414	134	351	914	1067	2000	0
**15-17**	Food	345	98	287	789	952	2600	0
	+ mandatorily fortified food	394	133	332	862	1026	2600	0
	+ voluntarily fortified food	401	134	338	873	1033	2600	0
	+ supplements	421	141	355	927	1099	2600	0
**18-39**	Food	366	104	305	837	1009	3000	0
	+ mandatorily fortified food	421	143	361	902	1068	3000	0
	+ voluntarily fortified food	424	145	364	907	1070	3000	0
	+ supplements	447	149	380	974	1161	3000	0
**40-64**	Food	432	124	359	983	1184	3000	0
	+ mandatorily fortified food	537	195	468	1112	1322	3000	0
	+ voluntarily fortified food	537	194	468	1112	1315	3000	0
	+ supplements	553	198	481	1155	1353	3000	0

(1)The UL for retinol is 800 µg/day in children aged 1-3 years and 1100 µg/day in children aged 4-6 years old. UL: upper intake level

Table S3. Usual intake of retinol (µg/day) from food, fortified food and supplements in Belgian women (3-64 years), Belgian study on the intake of vitamins A, D, E and K (VITADEK-study) 2015

Gender/age		Usual intake	Percentiles 5	50	95	97.5	UL	% >UL
**WOMEN**								
	Food							
**3-6**		262	89	231	539	628	800-1100(1)	0
	+ mandatorily fortified food	304	120	272	598	686	800	0
	+ voluntarily fortified food	310	123	279	600	692	800	0
	+ supplements	343	127	301	713	844	800	1
**7-10**	Food	266	91	235	546	635	1500	0
	+ mandatorily fortified food	304	119	274	590	687	1500	0
	+ voluntarily fortified food	315	123	282	615	724	1500	0
	+ supplements	331	125	292	669	794	1500	0
**11-14**	Food	269	92	238	553	643	2000	0
	+ mandatorily fortified food	308	120	278	599	700	2000	0
	+ voluntarily fortified food	312	124	280	612	696	2000	0
	+ supplements	332	127	295	672	793	2000	0
**15-17**	Food	272	94	241	559	650	2600	0
	+ mandatorily fortified food	310	121	280	591	678	2600	0
	+ voluntarily fortified food	316	124	284	616	708	2600	0
	+ supplements	339	128	297	695	829	2600	0
**18-39**	Food	284	98	252	582	676	3000	0
	+ mandatorily fortified food	330	131	298	635	728	3000	0
	+ voluntarily fortified food	341	133	305	664	770	3000	0
	+ supplements	407	141	333	932	1190	3000	0
**40-64**	Food	306	107	271	622	722	3000	0
	+ mandatorily fortified food	372	151	338	707	807	3000	0
	+ voluntarily fortified food	380	154	343	722	838	3000	0
	+ supplements	430	164	374	905	1063	3000	0

(1) The UL retinol for is 800 µg/day in children aged 1-3 years and 1100 µg/day in children aged 4-6 years old. UL: upper intake level

Table S4. Usual intake of vitamin A (µg/day) from food, fortified food and supplements in Belgian men (3-64 years), excluding miss-reporters, Belgian study on the intake of vitamins A, D, E and K (VITADEK-study) 2015

Gender/age		Usual intake (CI)	Percentiles 5	50	95	97,5	EAR	% < EAR	UL	% > UL
3-6	Food	653	257	578	1299	1517	205-245[(1)]	3	800-1100[(1)]	1
	+ mandatorily fortified food	691	289	618	1344	1552		2		3
	+ voluntarily fortified food	721	311	649	1371	1605		1		3
	+ supplements	736	313	662	1399	1618		1		4
7-10	Food	678	267	601	1351	1577	320	10	1500	0
	+ mandatorily fortified food	721	304	648	1378	1614		6		0
	+ voluntarily fortified food	749	318	676	1431	1642		5		0
	+ supplements	754	321	683	1429	1643		5		1
11-14	Food	705	278	625	1404	1639	580	44	2000	0
	+ mandatorily fortified food	753	320	675	1448	1711		37		0
	+ voluntarily fortified food	770	334	698	1472	1696		35		0
	+ supplements	797	337	717	1530	1785		32		0
15-17	Food	729	288	646	1452	1696	570	40	2600	0
	+ mandatorily fortified food	786	337	701	1526	1753		32		0
	+ voluntarily fortified food	801	351	726	1516	1741		30		0
	+ supplements	835	353	746	1628	1874		28		0
18-39	Food	829	324	733	1658	1939	570	31	3000	0
	+ mandatorily fortified food	881	369	787	1710	1983		24		0
	+ voluntarily fortified food	895	382	801	1729	2009		23		0
	+ supplements	918	385	820	1791	2080		22		0
40-64	Food	1023	399	904	2048	2396	570	18	3000	0
	+ mandatorily fortified food	1164	503	1045	2225	2576		9		0
	+ voluntarily fortified food	1175	501	1053	2250	2609		9		0
	+ supplements	1188	510	1073	2258	2610		8		0

[(1)]The EAR for vitamin A is 205 µg/day in children aged 1-3 years and 245 µg/day in children aged 4-6 years old. The UL for vitamin A (retinol) is 800 µg day in children aged 1-3 years and 1100 µg/day in children aged 4-6 years old.
EAR: Estimated average requirement; UL: upper intake level

Table S5. Usual intake of vitamin A (µg/dag) from food, fortified food and supplements in Belgian women (3-64 years), excluding miss-reporters, Belgian study on the intake of vitamins A, D, E and K (VITADEK-study) 2015

Gender/age		Usual intake (CI)	Percentiles 5	50	95	97,5	EAR	% < EAR	UL	% UL
3-6										
	Food	607	270	552	1129	1295	205-245[(1)]	3	800-1000[(1)]	0
	+ mandatorily fortified food	651	311	598	1181	1338		1		0
	+ voluntarily fortified food	674	322	620	1212	1383		1		0
	+ supplements	710	327	651	1302	1473		1		1
7-10	Food	628	279	571	1167	1338	320	9	1500	0
	+ mandatorily fortified food	673	321	619	1219	1370		5		0
	+ voluntarily fortified food	693	326	635	1247	1415		5		0
	+ supplements	718	334	659	1312	1469		4		0
11-14	Food	648	289	590	1206	1383	490	33	2000	0
	+ mandatorily fortified food	691	329	636	1252	1414		26		0
	+ voluntarily fortified food	705	331	651	1262	1434		23		0
	+ supplements	729	342	671	1327	1543		22		0
15-17	Food	667	297	607	1241	1423	490	31	2600	0
	+ mandatorily fortified food	716	334	652	1311	1500		24		0
	+ voluntarily fortified food	721	341	668	1291	1445		23		0
	+ supplements	741	343	676	1360	1559		21		0
18-39	Food	744	329	676	1389	1595	490	23	3000	0
	+ mandatorily food	799	372	732	1450	1657		16		0
	+ voluntarily fortified food	812	380	743	1479	1682		15		0
	+ supplements	869	390	782	1645	1914		14		0
40-64	Food	891	394	809	1664	1911	490	13	3000	0
	+ mandatorily fortified food	986	465	906	1786	2043		6		0
	+ voluntarily fortified food	995	467	912	1798	2061		6		0
	+ supplements	1055	489	969	1910	2165		5		0

(1) The EAR for vitamin A is 205 µg/day in children aged 1-3 years and 245 µg/day in children aged 4-6 years old. The UL for vitamin A (retinol) is 800 µg/day in children aged 1-3 years and 1100 µg/day in children aged 4-6 years old.

EAR: Estimated average requirement; UL: upper intake level.

Table S6. Usual intake of vitamin D (μg/day) from food, fortified food and supplements in Belgian men (3-64 years), excluding miss-reporters, Belgian study on the intake of vitamins A, D, E and K (VITADEK-study) 2015

Gender/age		Usual intake	Percentiles 5	50	95	97,5	AI	% Inadequate[(1)]	UL	% > UL
3-6	Food	2.10	0.78	1.86	4.20	4.88	15	ns[(2)]	25	0
	+ mandatorily fortified food	2.54	1.12	2.31	4.73	5.47	15	ns	25	0
	+ voluntarily fortified food	3.57	1.53	3.30	6.53	7.33	15	ns	25	0
	+ supplements	11.36	1.68	3.83	14.25	72.07	15	ns	25	3.43
7-10	Food	2.88	1.18	2.60	5.53	6.36	15	ns	25	0
	+ mandatorily fortified food	3.28	1.49	3.01	5.90	6.81	15	ns	25	0
	+ voluntarily fortified food	4.06	1.82	3.76	7.30	8.27	15	ns	25	0
	+ supplements	5.50	1.91	4.06	9.07	11.81	15	ns	25	0.48
11-14	Food	3.20	1.32	2.90	6.11	7.02	15	ns	50	0
	+ mandatorily fortified food	3.60	1.63	3.29	6.64	7.59	15	ns	50	0
	+ voluntarily fortified food	4.22	1.91	3.88	7.52	8.53	15	ns	50	0
	+ supplements	5.37	2.00	4.11	8.68	10.73	15	ns	50	0.34
15-17	Food	3.37	1.40	3.05	6.41	7.36	15	ns	50	0
	+ mandatorily fortified food	3.78	1.72	3.49	6.73	7.80	15	ns	50	0
	+ voluntarily fortified food	4.21	1.93	3.91	7.56	8.58	15	ns	50	0
	+ supplements	4.56	2.04	4.13	8.40	9.70	15	ns	50	0.00
18-39	Food	3.62	1.51	3.28	6.88	7.90	15	ns	50	0
	+ mandatorily fortified food	4.11	1.88	3.80	7.44	8.45	15	ns	50	0
	+ voluntarily fortified food	4.46	2.03	4.11	8.05	9.15	15	ns	50	0
	+ supplements	8.93	2.10	4.36	10.35	19.01	15	ns	50	1.72

40-64	Food	3.78	1.58	3.43	7.18	8.23	15		ns	50	0
	+ mandatorily fortified food	4.93	2.33	4.56	8.81	9.94	15		ns	50	0
	+ voluntarily fortified food	5.15	2.44	4.79	9.11	10.26	15		ns	50	0
	+ supplements	5.68	2.48	4.92	9.74	11.25	15		ns	50	0.25

(1)When not enough evidence is available to set an EAR, an AI is set. The AI is based on the average intake of an apparently healthy population.

(2) When the mean intake of the population > the AI, the risk for inadequate intake is low. No statement (ns) can be formulated on the adequacy of vitamin D when the mean intake < AI.

EAR: Estimated average requirement; UL: upper intake level; AI: adequate intake

Table S7. Usual intake of vitamin D (µg/day) from food, fortified food and supplements in Belgian women (3-64 years), excluding miss-reporters, Belgian study on the intake of vitamins A, D, E and K (VITADEK-study) 2015

Gender/age		Usual intake	Percentiles 5	50	95	97,5	AI	% Inadequate[1]	UL	% > UL
3-6										
	Food	2.84	0.85	2.39	6.33	7.58	15	ns[2]	15	0
	+ mandatorily							ns		
	fortified food	3.12	1.13	2.70	6.57	7.73	15		25	0
	+ voluntarily							ns		
	fortified food	3.86	1.50	3.45	7.62	8.83	15		25	0
	+ supplements	12.1	1.7	4.0	14.3	44.7	15	ns	25	3.1
								ns		
7-10	Food	2.86	0.86	2.41	6.37	7.64		ns	15	0
	+ mandatorily							ns		
	fortified food	3.15	1.12	2.73	6.65	7.74	15		25	0
	+ voluntarily							ns		
	fortified food	3.77	1.46	3.37	7.34	8.73	15		25	0
	+ supplements	9.1	1.5	3.7	11.2	18.2	15	ns	25	2.0
								ns		
11-14	Food	2.88	0.87	2.42	6.42	7.69		ns	15	0
	+ mandatorily							ns		
	fortified food	3.19	1.16	2.77	6.69	7.90	15		50	0
	+ voluntarily							ns		
	fortified food	3.68	1.40	3.24	7.37	8.45	15		50	0
	+ supplements	5.4	1.4	3.5	8.9	11.3	15	ns	50	0.4
								ns		
15-17	Food	2.90	0.87	2.44	6.46	7.74			15	0
	+ mandatorily							ns		
	fortified food	3.24	1.13	2.78	6.96	8.21	15		50	0
	+ voluntarily							ns		
	fortified food	3.62	1.34	3.22	7.36	8.46	15		50	0
	+ supplements	6.9	1.4	3.5	9.4	13.9	15	ns	50	1.0
								ns		
18-39	Food	2.97	0.90	2.50	6.61	7.92			15	0
	+ mandatorily							ns		
	fortified food	3.45	1.24	3.00	7.18	8.51	15		50	0
	+							ns		
	voluntarilyfortified	3.84	1.45	3.41	7.68	8.97	15		50	0

	food									
	+ supplements	11.6	1.6	3.9	17.8	37.7	15	ns	50	2.2
								ns		
40-64	Food	3.09	0.94	2.60	6.87	8.22		ns	15	0
	+ mandatorily							ns		
	fortified food	3.82	1.42	3.34	7.84	9.24	15		50	0
	+ voluntarily							ns		
	fortified food	4.24	1.67	3.77	8.38	9.79	15		50	0
	+ supplements	10.1	1.8	4.6	19.8	28.3	15	ns	50	1.4

(1) When not enough evidence is available to set an EAR, an AI is set. The AI is based on the average intake of an apparently healthy population.

(2) When the median intake of the population > the AI, the risk for inadequate intake is low. No statement (ns) can be formulated on the adequacy of vitamin D when the median intake < AI.

EAR: Estimated average requirement; UL: upper intake level; AI: adequate intake

Table S8. Usual intake of vitamin E (mg/day) from food, fortified food and supplements in Belgian men (3-64 years), excluding miss-reporters, Belgian study on the intake of vitamins A, D, E and K (VITADEK-study) 2015

Gender/age		Usual intake	Percentiles				AI[2]	% inadequate[2]	UL	% > UL
			5	50	95	97,5				
3-6	Food	7.20	3.68	6.76	12.23	13.67	6-9[1]	ns[3]	60	0
	+ fortified food	8.34	4.35	7.88	13.85	15.40	6	ns	60	0
	+ supplements	8.68	4.40	8.16	14.66	16.39	6	ic[4]	60	0
7-10	Food	10.16	5.51	9.62	16.66	18.51	9	ic	100	0
	+ fortified food	10.92	5.96	10.37	17.78	19.84	9	low	100	0
	+ supplements	11.14	6.06	10.58	18.29	20.22	9	low	100	0
11-14	Food	12.04	6.60	11.41	19.60	21.72	13	ns	120	0
	+ fortified food	12.74	7.04	12.12	20.54	22.90	13	ns	120	0
	+ supplements	12.99	7.22	12.29	21.23	23.40	13	ns	120	0
15-17	Food	13.16	7.25	12.48	21.36	23.64	13	ns	130	0
	+ fortified food	13.78	7.66	13.11	22.22	24.43	13	ic	130	0
	+ supplements	14.12	7.79	13.26	23.27	25.84	13	low	130	0
18-39	Food	14.49	7.99	13.75	23.50	26.04	13	ic	150	0
	+ fortified food	15.12	8.37	14.41	24.32	26.97	13	low	150	0
	+ supplements	15.60	8.49	14.74	25.58	28.46	13	low	150	0
40-64	Food	13.28	7.24	12.58	21.70	24.05	13	ic	150	0
	+ fortified food	14.65	7.98	13.91	23.81	26.31	13	ic	150	0
	+ supplements	15.29	8.10	14.35	25.63	29.06	13	ic	150	0

(1) The AI for vitamin E is 6 mg/day in children aged 1-3 years and 9 mg/day in children aged 4-6 years old.

(2) When not enough evidence is available to set an EAR, an AI is set. The AI is based on the average intake of an apparently healthy population.

(3) When the median intake of the population lies above the AI, the risk for inadequate intake is low. Since the distribution of requirements is not known no statement can be formulated on the adequacy of vitamin E when the median intake is lower than the AI.

(4) ic: inconclusive within a certain age-group

EAR: Estimated average requirement; UL: upper intake level; AI: adequate intake.

Table S9. Usual intake of vitamin E (mg/day) from food, fortified food and supplements in Belgian women (3-64 years), excluding miss-reporters, Belgian study on the intake of vitamins A, D, E and K (VITADEK-study) 2015

Gender/age		Usual intake	Percentiles 5	50	95	97,5	AI[2]	% inadequate[2]	UL	% > UL
3-6	Food	6.90	3.56	6.49	11.65	12.99	6-9[1]	ns[3]	60	0
	+ fortified food	7.71	4.06	7.29	12.80	14.17	6	ns	60	0
	+ supplements	8.29	4.17	7.72	14.24	16.13	6	ic[4]	60	0.00
7-10	Food	9.55	5.32	9.06	15.45	17.11	9	ic	100	0
	+ fortified food	10.27	5.75	9.76	16.51	18.24	9	low	100	0
	+ supplements	10.66	5.87	10.02	17.51	19.33	9	low	100	0.00
11-14	Food	10.73	6.01	10.19	17.28	19.12	11	ns	120	0
	+ fortified food	11.36	6.37	10.80	18.18	19.94	11	ic	120	0
	+ supplements	11.66	6.44	11.08	19.14	21.23	11	ic	120	0.00
15-17	Food	11.23	6.30	10.67	18.07	19.99	11	ns	130	0
	+ fortified food	11.77	6.71	11.17	18.76	20.66	11	ic	130	0
	+ supplements	11.96	6.74	11.41	19.31	21.28	11	low	130	0.00
18-39	Food	11.27	6.32	10.71	18.14	20.07	11	ns	150	0
	+ fortified food	11.94	6.72	11.38	19.12	21.15	11	low	150	0
	+ supplements	13.72	6.97	12.04	22.75	27.17	11	low	150	0.21
40-64	Food	10.15	5.66	9.64	16.39	18.16	11	ns	150	0
	+ fortified food	11.03	6.12	10.49	17.79	19.65	11	ic	150	0
	+ supplements	12.90	6.40	11.62	24.14	28.05	11	ic	150	0.00

[1] The AI for vitamin E is 6 mg/day in children aged 1-3 years and 9 mg/day in children aged 4-6 years old.

[2] When not enough evidence is available to set an EAR, an AI is set. The AI is based on the average intake of an apparently healthy population.

[3] When the median intake of the population lies above the AI, the risk for inadequate intake is low. Since the distribution of requirements is not known no statement can be formulated on the adequacy of vitamin E when the median intake is lower than the AI.

[4] ic: inconclusive within a certain age-group

EAR: Estimated average requirement; UL: upper intake level; AI: adequate intake.

Table S10. Usual intake of vitamin K (µg/day) from foods only in Belgian men (3-64 years), excluding miss-reporters, Belgian study on the intake of vitamins A, D, E and K (VITADEK-study) 2015

Gender/age	Usual intake	Percentiles				AI[2]	% inadequate[3]	UL	% > UL
		5	50	95	97,5				
3-6	59.1	20.98	51.05	124.11	147.14	12-20[1]	low[3]	200-300[1]	0.17
7-10	63.2	22.46	54.59	132.83	157.35	30	low	450	0.01
11-14	67.5	24.00	58.36	141.94	168.28	45	low	750	0.00
15-17	71.6	25.44	61.89	150.51	178.44	65	ns	900	0.00
18-39	89.7	31.17	77.11	190.73	226.76	70	ic[4]	1000	0.00
40-64	129.2	44.71	110.88	275.28	327.81	70	low	1000	0.00

[1] The AI for vitamin K is 12 µg/day in children aged 1-3 years and 20 µg/day in children aged 4-6 years old. The UL for vitamin K is 200 µg/day in children aged 1-3 years and 300 µg/day in children aged 4-6 years old.
[2] When not enough evidence is available to set an EAR, an AI is set. The AI is based on the average intake of an apparently healthy population.
[3] When the median intake of the population lies above the AI, the risk for inadequate intake is low. Since the distribution of requirements is not known no statement (ns) can be formulated on the adequacy of vitamin K when the median intake is lower than the AI.
[4] ic: inconclusive within a certain age-group.
EAR: Estimated average requirement; UL: upper intake level; AI: adequate intake.

Table S11. Usual intake of vitamin K (μg/day) from foods only in Belgian women (3-64 years), excluding miss-reporters, Belgian study on the intake of vitamins A, D, E and K (VITADEK-study) 2015

Gender/age	Usual intake	Percentiles				AI [2]	% Inadequate[3]	UL	% > UL
		5	50	95	97,5				
3-6	56.0	18.2	47.3	123.0	147.9	12-20[1]	low[3]	200-300[1]	0.2
7-10	64.8	21.1	54.8	142.1	170.8	30	low	450	0.0
11-14	72.5	23.6	61.3	158.9	190.6	45	low	750	0.0
15-17	78.8	25.7	66.7	172.7	207.3	65	low	900	0.0
18-39	101.4	32.4	85.3	224.6	270.3	70	ic[4]	1000	0.0
40-64	139.1	44.8	117.2	306.8	369.1	70	low	1000	0.0

(1) The AI for vitamin K is 12 μg/day in children aged 1-3 years and 20 μg/day in children aged 4-6 years old. The UL for vitamin K is 200 μg/day in children aged 1-3 years and 300 μg/day in children aged 4-6 years old.

(2) When not enough evidence is available to set an EAR, an AI is set. The AI is based on the average intake of an apparently healthy population.

(3) When the median intake of the population lies above the AI, the risk for inadequate intake is low. Since the distribution of requirements is not known no statement (ns) can be formulated on the adequacy of vitamin E when the median intake is lower than the AI.

(4) ic: inconclusive within a certain age-group

EAR: Estimated average requirement; UL: upper intake level; AI: adequate intake
